# What about microsaccades in the electroencephalogram of infants?

**DOI:** 10.1098/rspb.2016.0739

**Published:** 2016-07-27

**Authors:** Moritz Köster

**Affiliations:** Institute of Psychology, University of Münster, 48149 Münster, Germany

Kampis *et al*. [1] use gamma oscillations (here 25–35 Hz) in the scalp-recorded electroencephalogram (EEG) of infants to investigate the neuronal signatures of objects representations. Oscillations in the gamma range have been used in several infant studies in the recent years [2–4] and are viewed as an important tool to investigate preverbal object representation processes [5,6].

However, Kampis *et al*. [1] (and the authors of the other studies mentioned above [2–4]) do not mention that they tested for microsaccadic eye movements in their subjects, which is critical when analysing gamma-band oscillations in the EEG. In the EEG of adults, Yuval-Greenberg *et al.* [7] have demonstrated beyond doubt that microsaccadic eye movements contaminate the gamma-band activity measured by the EEG and are, like neuronal gamma-band responses, sensitive to different cognitive processes, including object representation processes. This and further studies also revealed that the approximately 20–90 Hz activity elicited by microsaccades has a much higher amplitude than gamma-band activity elicited by neural object representation processes [7–9]. Since then, in the adult literature, gamma-band activity in the EEG has been commonly reported after the removal of microsaccadic artefacts, for example with independent component procedures [8,9] (for a summary, see [10]). Importantly, these studies demonstrate that gamma-band activity can still be observed after the removal of microsaccadic artefacts. Thus, gamma-band analyses in the EEG remain a useful tool to investigate neural object representation processes in adults, when microsaccadic artefacts are removed [11,12], and may also serve to understand these processes in infants.

To conclude, in the study by Kampis *et al*. [1] and other recent EEG studies with infants [2–4] it is unclear whether microsaccadic eye movements are present and may contaminate the EEG signal, as in adults. The measured activity in the 25–35 Hz range may thus result from neuronal processes, eye movements or a mixture of both sources. I encourage the authors of this study and future studies investigating gamma-band oscillations in the infant EEG to clarify whether or not the EEG of infants is contaminated by similar microsaccadic eye movements as found in adults. Notably, if present, the characteristics of microsaccades of infants may differ from those in adults, and the development of age appropriate algorithms to remove microsaccadic artefacts in the EEG of infants is methodologically challenging. This would probably require the simultaneous application of EEG and eye-tracking methods, as well as relatively long periods of noise-free EEG recordings to run independent component analyses [7–9]. However, if microsaccadic artefacts should be present in the EEG of infants, only this would allow researchers to tear apart cognitive processes reflected in neural gamma-band oscillations and miniature eye movements, which possibly also reflect cognitive processes.
